# Chromatin features constrain structural variation across evolutionary timescales

**DOI:** 10.1073/pnas.1808631116

**Published:** 2019-01-18

**Authors:** Geoff Fudenberg, Katherine S. Pollard

**Affiliations:** ^a^Gladstone Institute of Data Science and Biotechnology, San Francisco, CA 94158;; ^b^Department of Epidemiology & Biostatistics, University of California, San Francisco, CA 94158;; ^c^Institute for Human Genetics, University of California, San Francisco, CA 94158;; ^d^Quantitative Biology Institute, University of California, San Francisco, CA 94158;; ^e^Institute for Computational Health Sciences, University of California, San Francisco, CA 94158;; ^f^Chan-Zuckerberg Biohub, San Francisco, CA 94158

**Keywords:** chromatin, evolution, comparative genomics, Hi-C, CTCF

## Abstract

The potential impact of structural variants includes not only the duplication or deletion of coding sequences, but also the perturbation of noncoding DNA regulatory elements and structural chromatin features, including topological domains (TADs). Structural variants disrupting TAD boundaries have been implicated both in cancer and developmental disease; this likely occurs via “enhancer hijacking,” whereby removal of the TAD boundary exposes enhancers to new target transcription start sites (TSSs). With this functional role, we hypothesized that boundaries would display evidence for negative selection. Here we demonstrate that the chromatin landscape constrains structural variation both within healthy humans and across primate evolution. In contrast, in patients with developmental delay, variants occur remarkably uniformly across genomic features, suggesting a potentially broad role for enhancer hijacking in human disease.

## Significance

Noncoding DNA sequences play crucial roles in gene regulation, including via three-dimensional genome organization where they define chromatin boundaries and segment the genome into a sequence of insulated neighborhoods. However, the relative importance of noncoding DNA elements, particularly in comparison with protein-coding DNA sequences, remains more poorly characterized. Here, we systematically test if chromatin boundary disruptions are under purifying selection. Our analyses uncover a genomewide depletion of structural variants that would have the potential to alter chromatin structure. This in turn has implications for predicting not only which variants are likely pathogenic in clinical genetics settings, but also which are likely key innovations in primate evolution, and argues for expanding the current gene-centric paradigm for interpreting structural variants.

Structural variants (1–3) cannot only disrupt coding sequences through deletion, duplication, or inversion, but can also perturb noncoding DNA regulatory elements, including enhancers and structural features of chromatin, with consequences in development and disease (4, 5). Chromatin boundaries at the borders of topologically associating domains [TADs (6, 7)] have recently garnered substantial interest for their structural and potential functional roles. Rather than specifying an intrinsically active or inactive state, TAD boundaries appear to both insulate physical contacts in 3D and block ectopic transcriptional activation between genomic elements on either side (7–10).

An emerging line of research implicates structural variants that alter TAD boundaries as functionally relevant in cancer (11–14). Given the functional insulation displayed by TAD boundaries, a likely mechanism is enhancer hijacking (15, 16), also previously termed “enhancer adoption” (17), whereby a structural variant removes or moves a TAD boundary to expose transcription start sites (TSSs) to regulatory enhancers from which they would normally be insulated. While there have been intriguing examples of TAD boundary disruptions in developmental diseases (18–21), the effect of structural variants on chromatin features like TAD boundaries has received relatively little systematic attention outside of cancer (22), until the past year (23–28).

**Fig. 1.**
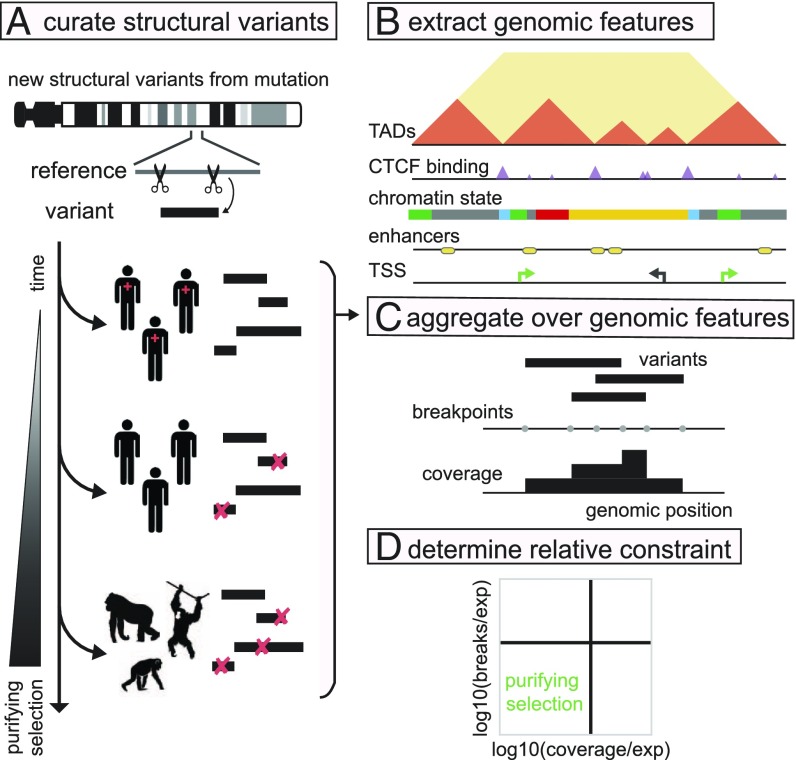
Approach to detect purifying selection against deleterious structural variants. (*A*) To study sets of structural variants subject to purifying selection for varying amounts of time, we obtained structural variants representing divergence with great apes (30), variation within the human population (31), and detected in patients (shown with red crosses) with developmental delay and autism (31). (*B*) To characterize the chromatin landscape, we curated: chromatin states, TSSs, CTCF binding clusters, and TAD boundaries. (*C*) We summarize each set of variants by their breakpoint frequency and coverage across the genome. (*D*) We then determine whether genomic features are relatively enriched or depleted for variant breakpoints and coverage. As structural variants subject to purifying selection are gradually removed from the population over time, we expect features under purifying selection to be depleted for breakpoint frequency and coverage.

To systematically test if TAD boundary disruptions are under purifying selection and compare their evolutionary constraint to that of other regulatory elements, we examined patterns of structural variation across evolutionary timescales from fixed differences between ape genomes to rare variants in human populations (Fig. 1). As the ability of negative selection to purge a given variant from the population depends on how deleterious it is and how much time selection has had to act on it (29), we can infer relative levels of evolutionary constraint on TAD boundaries by comparing the frequency with which they are altered by structural variants to that of other genomic elements and chromatin states. We find that deletions are strongly depleted at active chromatin states and TAD boundaries. This signature of negative selection is absent in patients with autism and developmental delay, where deletions occur remarkably uniformly across the genome, and in cancer, where deletions in fact show a slight enrichment for disrupting otherwise important features. Together our analyses uncover a genomewide pattern of negative selection against deletions that could potentially alter chromatin structure and lead to enhancer hijacking.

## Results

### Data and Methods.

To study structural variants subject to selection for different periods of time, we obtained sets representing divergence with great apes (30), variation within the human population (31), and those detected in patients with developmental delay and autism (31). For each dataset, we summarized overlap of structural variants with a given genomic feature two ways: breakpoint frequency (starts or ends in feature) and coverage (base pairs in feature) (Fig. 1). While related, these could in principle capture different factors; for example, a key genomic feature could be adjacent to a region prone to frequent breaks, yet be locally depleted for deletions that remove it. We focus on deletions, as duplications can either be in tandem, adjacent to the original copy, or elsewhere in the genome, adding additional complexity to their interpretation (22).

To characterize the chromatin landscape, we curated the following genomic features: chromHMM chromatin states from Roadmap (32), cross-tissue gene expression for TSSs from GTEx (33), TAD boundaries from high-resolution Hi-C data, called using an arrowhead score (34), and binding clusters for the insulator protein CTCF from ENCODE (35). CTCF frequently demarcates TAD boundaries (6, 7) and CTCF ChIP-seq data are currently available for a broader set of cell types than is high-resolution Hi-C data. We quantified the strength of a TSS in GTEx as the sum of its expression across human tissues, because consequences of a genetic variant on organismal fitness could arise from its impact on expression in any tissue. We considered two alternative ways to integrate expression data across tissues, depending on the primary impact on organismal fitness: the max, if fitness primarily depends on the tissue where a TSS is most highly expressed, and the Gini index, if fitness primarily depends on how stably TSSs are expressed across tissues. We found that both max (Spearman *R* = 0.94, *P* < 1 × 10^−10^) and Gini index (R = −0.74, *P* < 1 × 10^−10^) were highly correlated with the sum and gave qualitatively similar results. Similarly, we quantified the strength of a CTCF cluster as its aggregate binding across cell lines. TSSs and the midpoints of CTCF clusters were extended ±5 kb to enable consistent comparisons with TAD boundaries.

To quantitatively evaluate relative levels of purifying selection on different genomic features, it is critical to normalize deletion rates by their expected levels. We quantified this expectation as a uniform distribution across the genome, given the proportion of the genome covered by that genomic feature (*Methods*). We refer to a genomic feature with fewer variants than expected as “depleted.” Since we are unaware of detailed position-specific mutational models for germline structural variants, we emphasize that our approach assumes the mutation rate is fairly similar across genomic regions and features for a particular set of structural variants. Our analyses should be fairly robust to this simplifying assumption for two reasons. First, when comparing across different parts of the genome, we do not focus on absolute levels of depletion but rather difference in relative depletion between boundaries and other features. Second, we compare relative depletion of rare and common variants at boundaries, which controls for differences in mutation rates across genomic regions. This approach could be extended as it becomes feasible to model differences in structural variant mutation rates and patterns genomewide.

### Ape Deletions Are Strongly Depleted at Active Chromatin States.

**Fig. 2.**
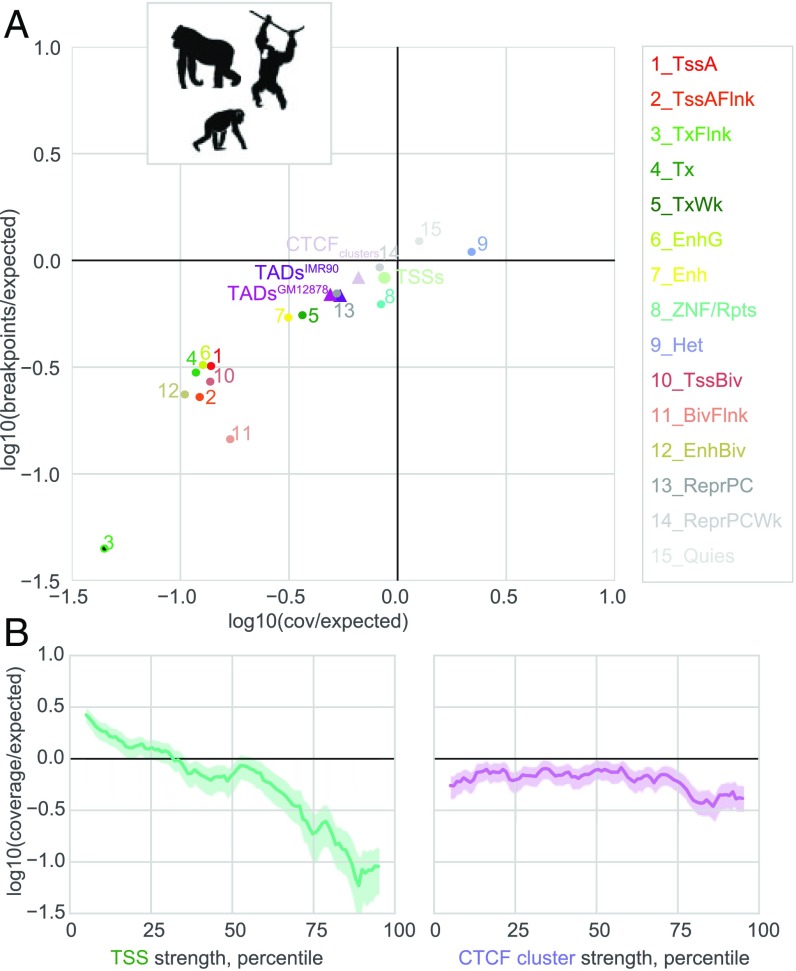
Ape deletions show patterns of purifying selection at active chromatin states, CTCF clusters, and TAD boundaries. (*A*) Deletions observed in apes (30) have relatively low coverage and breakpoint frequency in active genomic features and at TAD boundaries. Circles represent the average across 127 Roadmap cell types; see *SI Appendix*, Fig. S1*A* for variability of these estimates across cell types. Log10(observed/expected) represents deviations from a uniform distribution across the genome, accounting for the proportion of the genome covered by a given genomic feature (*Methods*). State 3 had no observed breakpoints or coverage and is shown with a black center at the minimal plotted *x*–*y* value, for display. (*B*) Ape deletion coverage at TSSs (*Left*) and CTCF clusters (*Right*) scales with the strength of these genomic features. Curves show average expected coverage as a function of feature strength in a sliding window (±5 percentiles); shaded areas represent 5th and 95th percentiles calculated over 1,000 bootstrap samples.

We first investigated the relationship between great ape deletions and human chromatin states. We considered 2,565 deletions relative to the human genome that were fixed in at least one ape species [(Bornean and Sumatran orangutans, any of four chimpanzee subspecies, bonobos, and Eastern and Western gorillas (30)] and were also parsimonious (i.e., not better explained by duplication in the human lineage). We found that both deletion breakpoints and coverage were depleted in active chromatin states (Fig. 2*A*), consistent with purifying selection acting to purge deletions affecting transcriptionally important portions of the genome. Indeed, only quiescent chromatin and heterochromatin were not consistently depleted for either coverage or deletion breakpoints across cell types (*SI Appendix*, Fig. S1*A*). TAD boundaries were also avoided by deletions, and avoided slightly more on average than TSSs. Confirming these observations, we found similar patterns for a more recently characterized set of gorilla deletions (36) (*SI Appendix*, Fig. S1).

We next examined if the strength of negative selection at TSSs and CTCF clusters relates to the strength of these features. Coverage was more depleted at more highly expressed TSSs (Fig. 2B), consistent with stronger purifying selection at more broadly important genes. Similarly, we found that both breakpoints and coverage were more depleted for stronger CTCF clusters (Fig. 2B and *SI Appendix*, Fig. S2). Collectively these findings argue that purifying selection acts to remove deleterious variants that would perturb functionally important chromatin features, including TAD boundaries, at the timescale of great ape evolution.

### Human Deletions Reveal Details of Selective Constraint Across Chromatin Features.

**Fig. 3.**
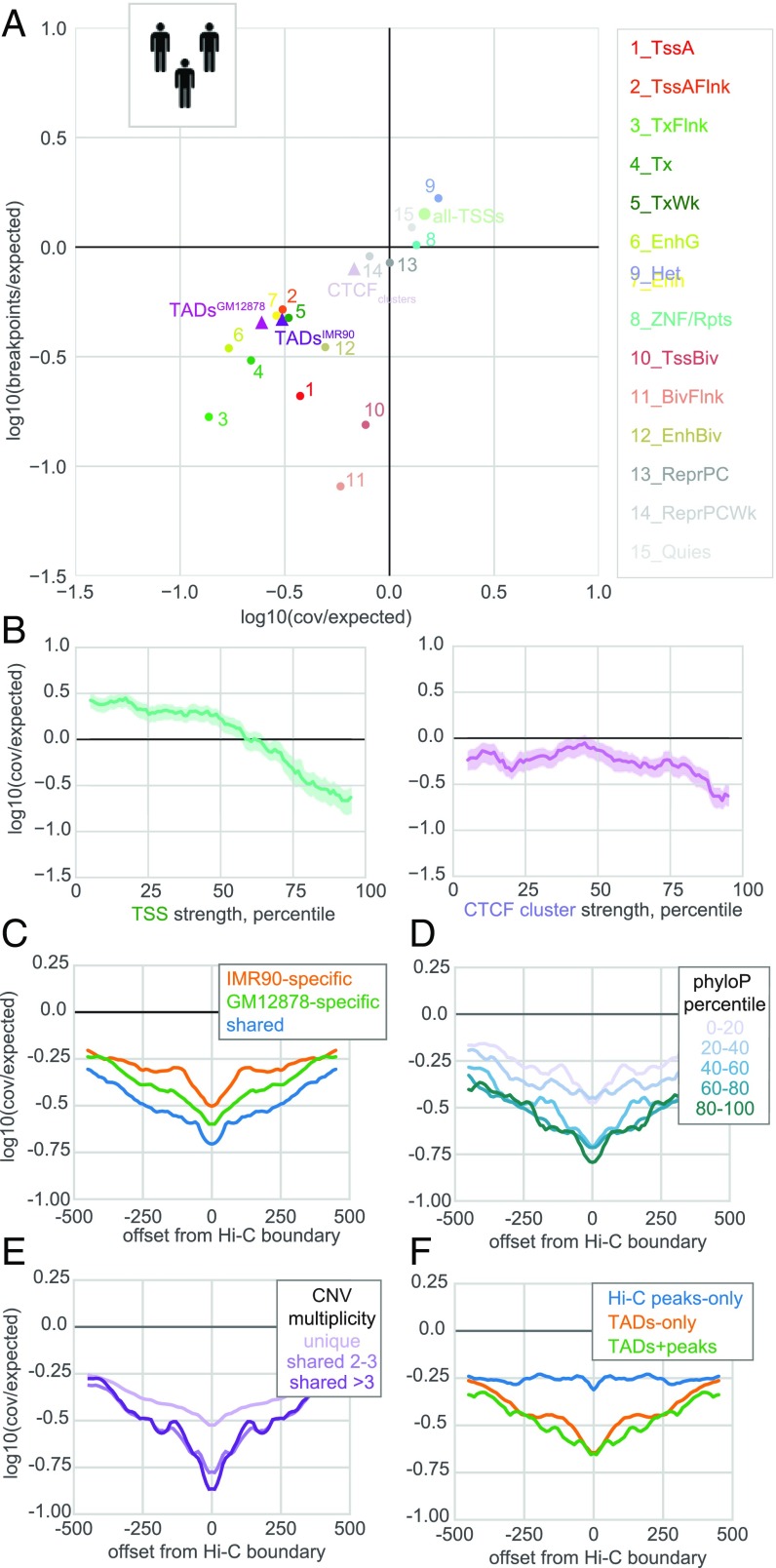
Human deletions reveal the spectrum of purifying selection across genomic features. (*A*) Deletions observed in healthy humans (31) have lower coverage and breakpoint frequency in active states and at TAD boundaries. Circles represent the average across 127 Roadmap cell types. Log10(observed/expected) represents deviations from a uniform distribution across the genome, as in Fig. 2 (*Methods*). (*B*) Healthy human deletion coverage at TSSs (*Left*) and CTCF clusters (*Right*) scales with the strength of these genomic features, plotted as in Fig. 2*B*. (*C*–*F*) Coverage in ±500-kb genomic region at 10-kb binned resolution. (*C*) TAD boundaries shared across cell types are more depleted for human deletions than those found in only one cell type. (*D*) TAD boundaries with more evolutionary conservation at the base-pair level are more depleted for deletions. (*E*) Deletions shared across individuals are more depleted at TAD boundaries. (*F*) TAD boundaries are more depleted for deletions than Hi-C peak bases. Note these and other curves approach zero at ∼5–10 Mb (*SI Appendix*, Fig. S3*H*).

We next investigated the connection between deletions found in healthy humans (31) and chromatin features (Fig. 3). These 20,089 deletions are segregating in the human population and generally have not been under selection for as long as deletions that are fixed differences between apes. Nevertheless, deleterious structural variants should be depleted in healthy adults. As observed for apes, human deletions were depleted in active chromatin states and at TAD boundaries (Fig. 3*A*), again arguing that purifying selection acts to purge deletions that would perturb TADs. We found similar, although less pronounced, patterns (*SI Appendix*, Fig. S1) in an independent set of human deletions from a smaller set of individuals (37), and note a similar depletion at TAD boundaries was reported for International Cancer Genome Consortium germline deletions (11). As for ape deletions, more highly expressed TSSs and stronger CTCF clusters were more depleted (Fig. 3*B*), arguing that the strength of purifying selection directly relates to the importance of a chromatin feature. As CTCF clusters were more avoided than TSS up to the ∼60th percentile of aggregate GTEx expression, these noncoding features could be as important as many coding features. Interestingly, CTCF motifs alone were not particularly depleted (*SI Appendix*, Fig. S3*A*), even after stratifying by motif quality (38), consistent with only a fraction being sufficiently occupied to enact structural and functional roles (39). These findings collectively argue that within the human population, purifying selection acts to remove deleterious variants that would perturb important chromatin features.

Leveraging the larger number of deletions in this dataset, we next investigated the coverage of deletions not only at TAD boundaries, but in the surrounding region as well (Fig. 3 *C*–*F*). This revealed that deletions are broadly depleted around TAD boundaries, and most depleted right at boundary sites. We additionally find that (*i*) boundaries called in multiple cell types are more depleted (∼1.4-fold for two versus only one cell type, Fig. 3*C*); (*ii*) boundaries with higher average basewise conservation are more depleted [∼2.2-fold more for the top versus bottom quintile of phyloP (40), Fig. 3*D*]; and (*iii*) deletions present in multiple people are more depleted at boundaries (∼1.7-fold, Fig. 3*E*), consistent with shared variants having spent more time under purifying selection.

Surprisingly, we found depletion at TAD boundaries showed little dependence on within-cell-type insulation (*SI Appendix*, Fig. S3*B*). This suggests the called set of boundaries all provide sufficient insulation to regulate genes in their neighborhoods, and weaker structural features may also play important functional roles. As boundaries might be most important when insulating genes with very different expression levels, we tested if boundaries over which GTEx expression is discordant show stronger signatures of deletion avoidance. Aggregating expression in a window on each side of a boundary for each tissue type, and taking the maximal difference between the two sides across tissue types, we found only weak evidence in support of this hypothesis (*SI Appendix*, Fig. S3*C*).

Since TSSs of active genes are avoided by deletions, we next tested if the depletion at TAD boundaries could result from their genomic proximity to actively expressed genes. When we stratified boundaries by their distance to the nearest highly expressed TSS, however, we found that depletion leveled out to genomewide average levels after ∼100 kb (*SI Appendix*, Fig. S3*D*). This argues that purifying selection can act on variants whose direct deleterious consequence is to perturb TAD boundaries.

Another notable feature of chromosome folding is focal peaks in Hi-C maps, associated with strong CTCF binding overlying oriented motifs in the corresponding cell type (often termed loops, ref. 34). We found, however, that TAD boundaries are more depleted than Hi-C peak bases (∼2.2-fold, Fig. 3*F*). Consistently, we found TAD boundaries are also more conserved at the single-nucleotide level than Hi-C peak bases, as measured by either their maximum or average phyloP score (*SI Appendix*, Fig. S3*E*). We note that the moderate depletion at Hi-C peak bases corresponds to that of the average cross-cell-type CTCF cluster strength at these peaks (−0.25, corresponding to the 85th percentile). Together this suggests TADs have broader, or more important, functional roles than peaks.

### Active Chromatin States and Chromatin Boundaries Are Disrupted in Patients with Developmental Delay or Autism.

**Fig. 4.**
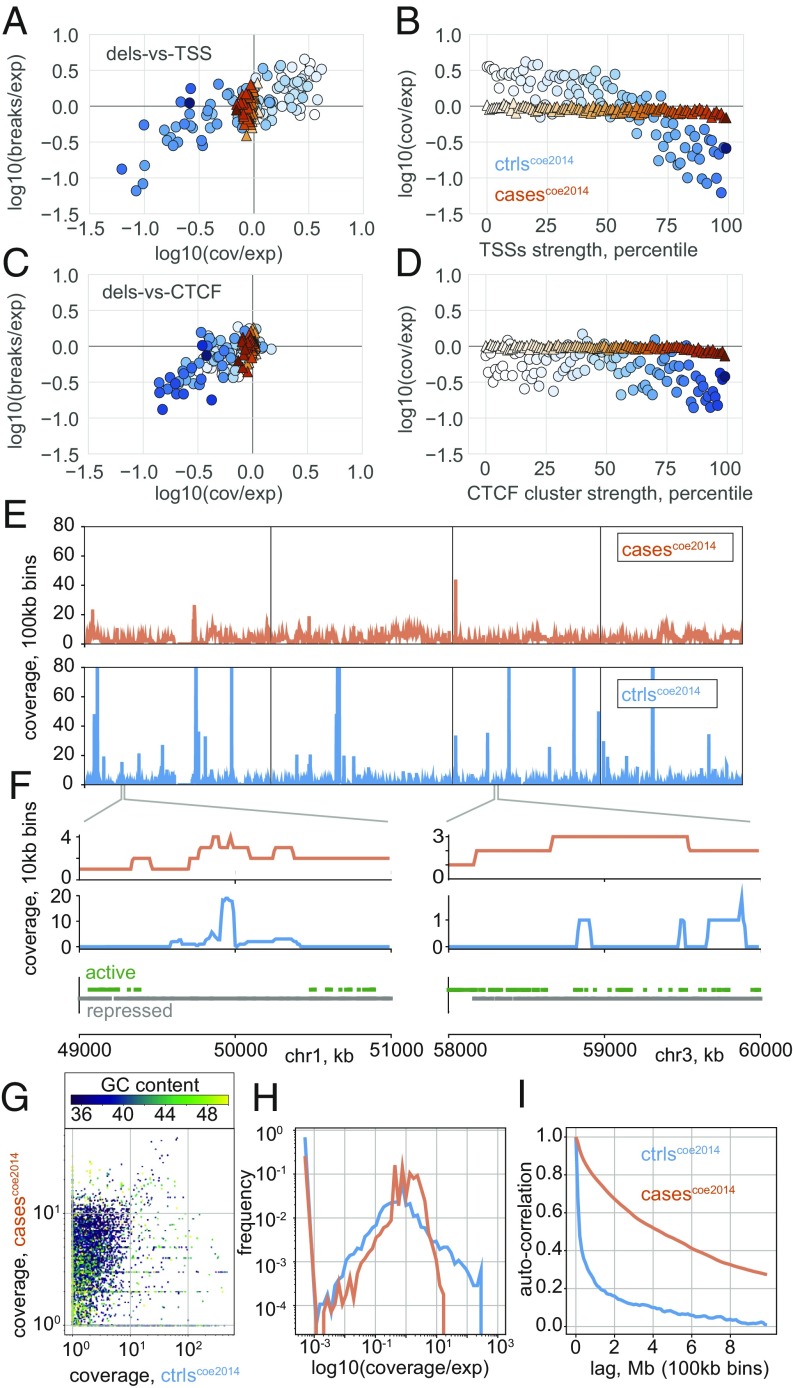
Deletions in human disease show little avoidance of key genomic features. (*A*) Average deletion coverage and breakpoint frequency for TSSs stratified and shaded by strength. Log10(observed/expected) represents deviations from a uniform distribution across the genome, as in Figs. 2 and 3 (*Methods*). Unlike for healthy subjects (blue), deletions from patients with developmental delay and autism (orange) show no avoidance of strong TSSs, either for coverage or breakpoint frequency, both cohorts from Coe et al. (31). (*B*) Deletion coverage in patients shows little relationship with TSS strength. (*C* and *D*) As for *A* and *B*, but for CTCF clusters. (*E*) Binned deletion coverage at 100 kb from patients (orange) and healthy controls (blue) across the first four chromosomes illustrate differences in their large-scale distribution across the genome. (*F*) Binned deletion coverage at 10 kb above tracks showing inactive (gray) versus active (green) Roadmap states. The region on chr1 with a long stretch of inactive states (*Left*) shows an island of high coverage in healthy subjects; the mixed states on chr3 (*Right*) show broadly elevated coverage in patients, compared with the more punctuated coverage in healthy subjects. (*G*) Binned deletion coverage at 100 kb, colored by GC content and shown on a log scale with a pseudocount of one for display, do not highly correspond for patients and healthy subjects. (*H*) Coverage per 100-kb bin shows more uniformity in patients. (*I*) Autocorrelation of 100-kb binned deletion coverage profiles varies more slowly in patients.

To investigate when purifying selection had little time to act, we considered 6,507 deletions in patients with developmental delay or autism (31). In contrast with deletions from apes and healthy humans, deletions in affected individuals displayed little avoidance of TSSs or CTCF clusters, regardless of the strength of these genomic features (Fig. 4 *A*–*D*). Consistently, active chromatin states and TAD boundaries showed no depletion in patients (*SI Appendix*, Figs. S1 and S3*F*).

In fact, deletions in patients display a remarkably uniform distribution across the genome (Fig. 4 *E*–*I*), in addition to being longer (31), as compared with deletions in healthy individuals. This is observed for deletions in patients both in the more slowly decreasing autocorrelation (Fig. 4*I*) and the less-skewed distribution (Fig. 4*H*) of the coverage profiles. We also note that the coverage profile of deletions in patients is not particularly correlated with that of controls (Fig. 4*G*).

To gain additional confidence in our observations of uniformity in patients with developmental delay and autism, we analyzed the 380,371 cancer deletions from COSMIC (41) with the same approach. We were motivated by previous reports linking cancer deletion frequency with chromatin state in the Pan-Cancer Analysis of Whole Genomes dataset (13). We found cancer deletions exhibited distinct patterns from those in either developmental delay patients or healthy controls (*SI Appendix*, Fig. S4). Indeed, as a function of increasing TSS or CTCF cluster strength, cancer deletions displayed an increasing enrichment for breakpoints, as well as for coverage of all but the longest quintile of deletions (*SI Appendix*, Fig. S4).

As genomewide analyses have been used to implicate specific boundary deletions in cancer etiology (14), we investigated properties of the boundaries deleted in the ape, healthy human, and developmental delay and autism patients. First, these cohorts differed in the gene ontology (GO) term enrichments of genes associated with recurrently deleted TAD boundaries: sensory perception in apes; immune-related in healthy humans; and chromatin-related in patients [using GO-rilla (42), Datasets S1 and S2]. Second, autism and developmental delay cases were moderately enriched for peaks of deletion coverage overlapping TAD boundaries compared with controls (*SI Appendix*, *Supplemental Text*). We refrained from determining the significance of individual deleted TAD boundaries, since the genomewide enrichment was low. Nevertheless, by visual inspection there are intriguing candidates for future analyses (*SI Appendix*, Fig. S5). Combined with our observations that disruptions to TAD boundaries are generally avoided in healthy humans, these results suggest that disruption of TAD boundaries could be a broad cause of disease beyond the known examples.

### Duplications Display a More Complex Relationship with Chromatin Features than Deletions.

We next considered how functional constraint influences the patterns of both duplications and deletions across evolutionary timescales. For a given level of average constraint on a class of genomic features, we expect structural variants to be most avoided for apes, then healthy humans, followed by humans with diseases, reflecting decreasing time for selection to have operated. This is indeed what we observe for deletions of TSSs, as would be expected if they were generally deleterious and under purifying selection (*SI Appendix*, Fig. S6*A*). Unexpectedly, CTCF clusters seem to be similarly, or even slightly less, avoided for deletions in apes compared with healthy humans (*SI Appendix*, Fig. S6*B*). For healthy humans, we observed similar, yet less-pronounced, patterns for duplications than for deletions. Interestingly, longer duplications were the main contributor to the remaining avoidance (*SI Appendix*, Fig. S7), which may indicate a greater importance of genomic context for duplications relative to deletions. Surprisingly, ape duplications show no clear trend for TSSs or CTCF clusters, which held after stratifying by length (*SI Appendix*, Fig. S7), in contrast to duplications in healthy humans. However, we note that ape duplications are on average much shorter than those in healthy humans, and the shortest human duplications also show little avoidance of TSSs or CTCF clusters. Additionally, few ape duplications remain after filtering (1,175), making the lack of signal inconclusive. As synteny breakpoints are avoided within TADs (25, 26), our observations argue that the details of how a structural variant impacts genomic organization can determine its effect on fitness.

## Discussion

In summary, we find evidence for purifying selection acting on structural variants, depending on their local chromatin context. Not only are deletions depleted in active chromatin states both in apes and the human population, but also at CTCF sites and TAD boundaries. Indeed, boundaries are avoided as strongly as intermediately expressed TSSs, suggesting parts of the coding and noncoding genome could be equally important from the point of view of deletions. In contrast with these sets of variants that had time to experience purifying selection, we found that variants present in patients with autism and developmental delay were surprisingly uniform across chromatin states, and displayed no preferential avoidance of strongly expressed TSSs or strongly bound CTCF sites.

The relatively indiscriminate disruption of the genome by deletions in patients with developmental delay and autism was unexpected. One potential reconciliation comes with our observation that deletions observed once were much less depleted at TAD boundaries than shared deletions (Fig. 3*E*). If developmental delay and autism deletions are largely de novo, and reflect the mutation pattern, whereas most control deletions have survived some negative selection, this could partially explain the apparent lack of avoidance at otherwise important chromatin features. Larger cohorts or studies specifically designed to assay de novo variants in healthy humans will be necessary to better untangle the allele frequency spectrum and test this hypothesis. Another point to note is that patients show deletions with sizes that are never seen in healthy people (31). Two nonexclusive possibilities are that many of these deletions directly contribute to developmental disease or that they arise by a different mutational process. We further note that the pattern for deletions from those of developmental delay patients differs from the pattern seen for cancer deletions, which actually display enrichment for breakpoints at highly expressed TSSs and CTCF clusters. We speculate this may either stem from different mutational mechanisms for somatic alterations in cancers compared with deletions in autism and developmental delay patients, including transcription-related mutagenesis for deletions in cancer, or widespread positive selection for deletions in cancer genomes.

While we find evidence for purifying selection acting on structural variants that would alter chromatin boundaries in apes as well as in healthy humans, an important caveat to our present study is that all analyses were conducted relative to the human genome due to the much greater quantity of human epigenome and chromatin conformation data (for review, see ref. 43). For example, our methodology might underestimate the relative depletion of structural variation at boundaries across evolutionary timescales if the exact positions of TAD boundaries are more dynamic over evolutionary time relative to other chromatin features. As broader characterizations of ape epigenomes and chromatin conformation become available, it will be interesting to revisit these analyses, potentially by inferring the set of structural features present in an ancestral ape.

Our findings further argue that structural variants with the potential to alter enhancer–promoter communication are under purifying selection. Interestingly, the overall distribution of both deletions and duplications in healthy humans rapidly plummets after ∼2 Mb (31), which is also roughly the furthest distance over which enhancers are known to act (4), the size of the largest TADs (44), and the distance over which cohesin enriches contact frequency (45). Put another way, it appears that deletions or duplications bringing genomic elements together that would otherwise never communicate are particularly avoided, suggesting it may be imperative to avoid enhancer hijacking. Supporting this hypothesis, very broadly expressed genes tend to be closer to very broadly bound CTCF sites (*SI Appendix*, Fig. S3*G*), consistent with a fundamental role of CTCF in constraining ectopic expression (46, 47) over evolutionary timescales (48). While mechanistic insights into TAD boundaries make us favor a role in preventing enhancer hijacking, it is also possible that TAD boundaries help guide enhancers toward target promoters, and that such additional roles could also contribute to negative selection on deletions at TAD boundaries.

Our results are also consistent with emerging mechanistic insights into enhancer–promoter communication (49). Our finding that Hi-C peaks are less avoided by deletions than TAD boundaries raises the possibility that TAD boundaries may generally have either broader, or more important, functional roles than Hi-C peaks. If enhancer–promoter contacts are very dynamic (50, 51) and enhancers are promiscuous (52) it may be relatively more important to keep enhancers from ectopically activating genes rather than specifying very specific enhancer–promoter pairings. Alternately, boundaries may be more important if they are more stable across cell types, and orchestrate different sets of peaks in different cell types.

An important caveat for using structural variants to assay functional importance of different genomic regions is the nonuniformity of the genome. Indeed, active regulatory elements are clustered along the genome (35, 53), making it difficult to discern their independent importance when structural variants can span multiple genomic features. Nevertheless, this property of structural variants can be beneficial for characterizing the chromatin landscape if disruptions of multiple elements, e.g., bound CTCF sites, are required to alter the boundary activity of TADs, as at the *HoxD* locus (54).

Collectively, our findings that TAD boundaries and strong CTCF sites display stronger purifying selection than many low-expressed coding sequences argue for rethinking the gene-centric paradigm of interpreting structural variants.

## Methods

### Structural Variant Datasets.

Great ape deletions (30) were filtered to require their being fixed in at least one of the assayed ape species (Bornean and Sumatran orangutans, any of four chimpanzee subspecies, bonobos, and Eastern and Western gorillas), not present in the hominid lineage (Hde), and not more parsimoniously explained by alteration in the human lineage. Deletions from healthy humans and patients with intellectual disability or developmental delay represent data from 11,256 controls and 29,083 patients (31). Cancer variants were obtained from COSMIC (ref. 41, release v84), representing duplication and deletion calls for 14,968 tumors. We used liftOver to convert ape variants from ref. 30 from hg18 to hg19 coordinates, and to convert COSMIC variants from hg38 to hg19. All other datasets had hg19 coordinates available. We also analyzed variants from refs. 36 and 37. We used unique combinations of start and end points to determine shared variants in the population. We limited all analyses to autosomes. For dataset statistics, see Dataset S3.

### Chromatin and Expression Datasets.

Chromatin state analyses were performed using the core 15-state model across 127 cell types from Roadmap (32). For display in Fig. 4*F*, Roadmap states were consolidated into inactive (grey, 8_ZNF/Rpts, 9_Het, 13_ReprPC, 14_PeprPCWk, 15_Quies) and active (green, other states). TSS analyses were performed using GTEx v6 release (33), where the strength of a TSS in GTEx was quantified as the sum of its expression across tissues. TAD boundary and Hi-C peak analyses were performed using published arrowhead domains and hiccups loop lists (34). CTCF binding clusters were obtained by downloading narrowPeak files from ENCODE (35) for the Broad center, and then using bedtools cluster on the aggregated set with a merge distance of 5 kb. We quantified the strength of a CTCF cluster as its aggregate binding across samples. TSSs and the midpoints of CTCF clusters were extended ±5 kb to enable consistent comparisons with TAD boundaries.

### Relative Abundance of Structural Variants.

For breakpoint frequency, the observed/expected was calculated as $\left(\sum_{i\in k}N_{i}\right)/\left(N_{total}\sum_{i\in k}\frac{S_{i}}{S_{total}}\right)$, where *i* indexes genomic regions within a particular feature class *k* (e.g., chromatin state, or quantile of CTCF binding strength), *S*_*i*_ is the size of region *i*, *N*_*i*_ is the number of variant breakpoints in region *i*, *S*_*total*_ is genome size, and *N*_*total*_ is the number of variant breakpoints genomewide. The observed/expected for coverage was calculated similarly, except with *N*_*i*_ and *N*_*total*_ counting base pairs covered by variants. Intersection of variant positions and genomic features was performed using bedtools (55). The first and last 2 Mb of each chromosome, and 2 Mb adjacent to centromeric regions (defined by UCSC hg19 gap file) were excluded from analysis, as these may be more prone to variant artifacts (31).

### Bootstrap Estimates for Coverage Versus Feature Strength.

To generate bootstrap estimates for the mean coverage as a function of feature strength, we sampled from the full list of observed (feature strength, coverage) pairs with replacement. We then computed averages in sliding windows of ±5 percentiles, and displayed the area between the 5th and 95th percentiles of mean values over 1,000 bootstrap samples.

## Supplementary Material

Supplementary File

Supplementary File

Supplementary File

Supplementary File
