# Supplementary material for: Chromatin features constrain structural variation across evolutionary timescales
Source: Proc Natl Acad Sci U S A. 2019 Jan 18;116(6):2175–80. doi: 10.1073/pnas.1808631116 (PMC6369792; doi:10.1073/pnas.1808631116)
Supplement: Supplementary File [file pnas.1808631116.sapp.pdf]

Supplemental Information for:
Chromatin features constrain structural variation across evolutionary timescales

Authors:
Geoff Fudenberg, Katherine S. Pollard

Emails: geoff.fudenberg@gladstone.ucsf.edu, katherine.pollard@gladstone.ucsf.edu

Contents:

Supplementary Text
Figs. S1 to S7
Captions for Datasets S1-S3
Table S1
References for SI reference citations

www.pnas.org/cgi/doi/10.1073/pnas.1808631116

Supplementary Text

As genome-wide analyses have been used to implicate specific boundaries deletions in cancer (1), we investigated whether this might be possible for deletions from patients with developmental delay or autism (2). To explore this, we permuted positions of deletions, calculated coverage profiles of these permuted events, and used these profiles to determine a threshold for recurrently deleted 10kb regions separately for cases and controls, using the 99.9th percentile of the respective permuted coverage profile. We found that such recurrently deleted 10kb regions were enriched at boundaries in cases, relative to controls (Fisher's exact test, OR 1.47, p-value <1e-4, **Table S1**).

To determine possible functional roles of deleted boundaries, we considered the enrichment of gene ontology categories for genes around TAD boundaries that were recurently deleted for the ape, healthy human, and developmental disease deletions using GO-rilla (3). These three gene sets displayed different GO term enrichments: ape deletions had terms related to sensory perception; healthy humans had immune-related terms; and developmental disease deletions had chromatin-related terms (**Dataset S1**). We note these results for genes near recurrently deleted TAD boundaries in apes agree with gene-based approaches that report recurrent deletion of olfactory perception loci across apes (4).

We then reasoned that local maxima, or peaks, in the genome-wide deletion coverage profile that overlap particular TAD boundaries could strengthen the case for a given boundary's putatively causal role in disease. A similar approach has been used for implicating particular genes from somatic copy alterations in cancer (5). We found that peaks in the coverage profiles were moderately enriched (OR 2.36, p-value .00610, **Table S1**). Since this genome-wide enrichment was relatively mild, we refrained from determining the significance of individual boundary elements in this patient cohort. Indeed, a challenge of using patient deletions to determine the role of individual TAD boundaries is that deletions in the disease cohort are particularly large (2), making it difficult to ascribe a role that primarily relates to disrupting the integrity of 3D genomic folding.

Nevertheless, by visual inspection there are intriguing candidates for future analyses, including a highly focally deleted boundary on chromosome 18 that appears to insulate a TAD containing the RNA-binding protein MEX3C from an adjacent TAD containing the gene DCC, involved in neurogenesis (**Fig. S5**). Combined with our observations that disruptions to TAD boundaries are generally avoided in healthy human cohorts, these results indicate that disruption of TAD boundaries could play important roles in diseases outside of those established in cancer.

Supplementary Methods

Permutation analysis for boundary deletions. To generate coverage profiles for permuted deletions, we used bedtools shuffle with hg19 autosomes as the genome, and the same excluded regions for the enrichment analyses above. For each set of variants we then took the 99.9th percentile of the coverage profile as a threshold to identify recurrently deleted regions. We then tabulated the number of recurrently deleted regions that overlapped TAD boundaries from GM12878 data versus those that were in other places in the genome and performed a Fisher's exact test on the resulting 2x2 table. To identify peaks in the coverage profiles we used the peakdet algorithm (Billauer E (2012). peakdet: Peak detection using MATLAB, http://billauer.co.il/peakdet.html), where the minimum required prominence is the same variant set specific threshold calculated above. We considered a peak as intersecting a TAD boundary if it was within +/- 10kb (i.e. one bin).

Enrichment of GO terms for genes around TAD boundaries. To quantify enrichment of GO terms around recurrently deleted TAD boundaries, we used GO-rilla (3) (http://cbl-gorilla.cs.technion.ac.il/) to calculates enrichments for a set of target genes versus a background set (6). We determined recurrently deleted boundaries as GM12878 TAD boundaries (7) with an observed coverage by deletions that exceeded the 99.9th percentile of permuted coverage profiles, calculated separately using 1000 permutations for each set of deletions. We took all TSSs with non-zero GTEx expression +/- 500kb around each recurrently deleted boundary as the three different target sets, and the background set as all TSSs +/- 500kb from any boundary (for lists of GO-rilla inputs **Dataset S2**). We note GO-rilla has annotations for only 45% of the TSSs on these input lists, as many gencode-V6 TSSs are un-annotated for non-protein-coding transcripts.

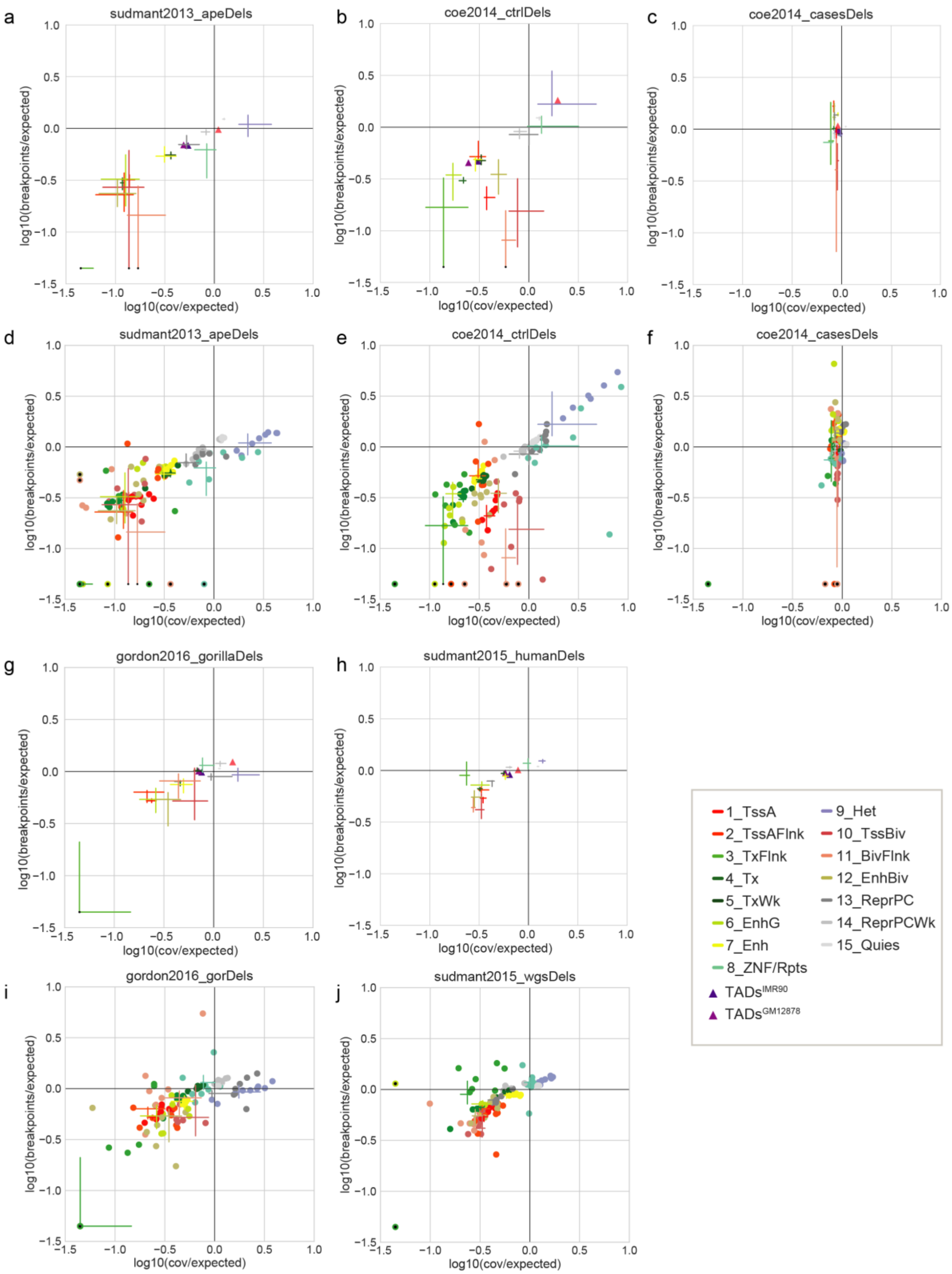

Fig. S1. (caption on next page)

Fig. S1.
a-c, g-h. Deletions observed in apes and healthy human have both lower coverage and breakpoint frequency than expected in active genomic features and at TAD boundaries. Crosses show the 25th and 75th percentiles across Roadmap cell types. A black endpoint indicates that no variants were observed for that chromatin class in the 25th percentile cell type, and the corresponding bar was truncated for display. Dataset for deletions is indicated above the associated plot. Note gorilla deletions (**g, i**) from (8) show similar patterns to ape deletions (**a,d**). Also note human deletions from (Sudmant et al., 2015) (**h,j**) show similar, though less pronounced, patterns as compared with healthy humans from (Coe et al., 2014) (**b,e**).
d-f, i-j. Crosses as in other panels, points represent individual Roadmap ESC cell types. Note that this represents 8 (E002, E008, E001, E015, E014, E016, E003, E024) of the 127 consolidated epigenomes in the Consolidated_EpigenomeIDs_summary_Table (jul2013.roadmapData.qc). Cell types with either no observed deletion coverage or breakpoints shown with a black center at the minimal plotted x- or y- value, for display.

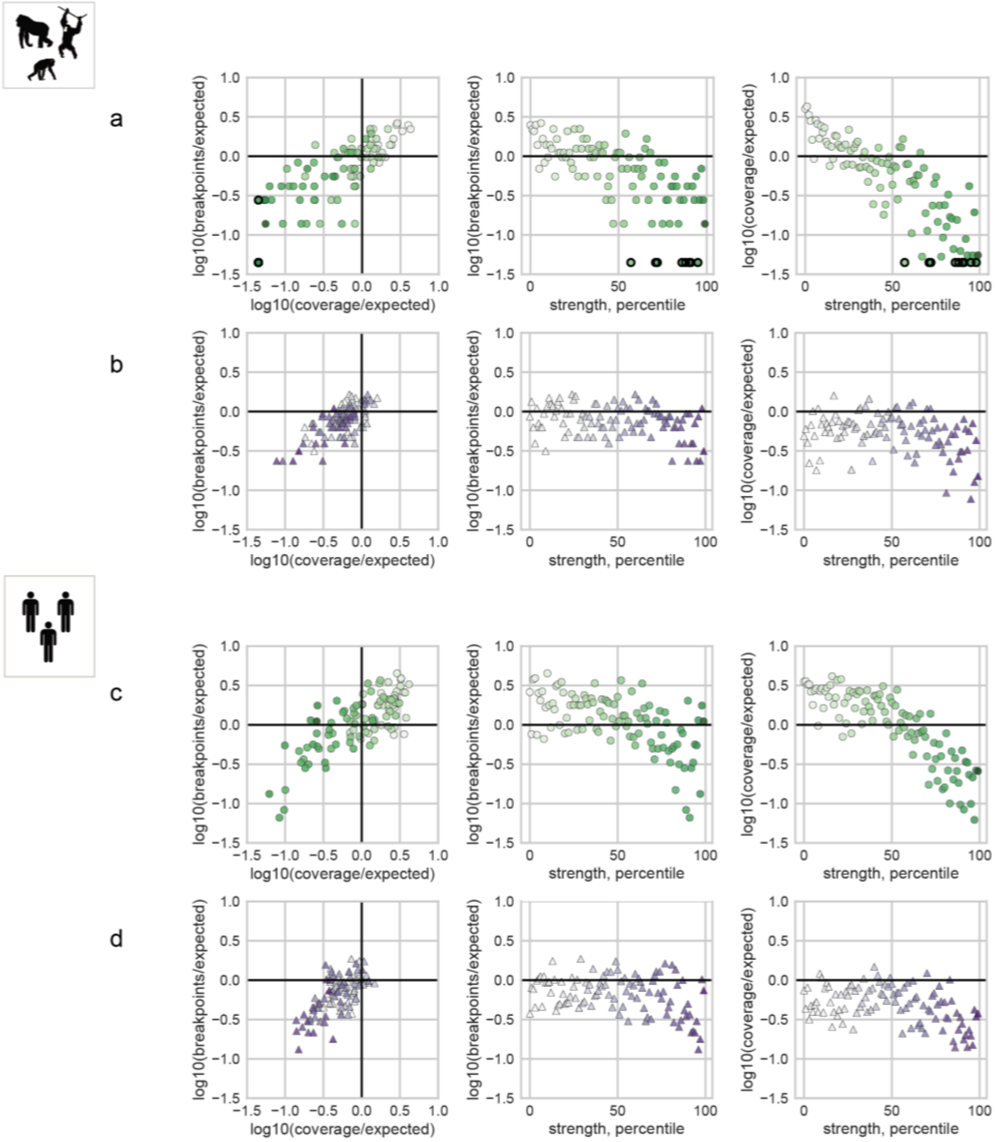

Fig. S2.
a. Ape deletion coverage and breakpoint frequencies at TSSs.
b. Ape deletion coverage and breakpoint frequencies at CTCF clusters.
c. Healthy human deletion coverage and breakpoint frequencies at TSSs.
d. Healthy human deletion coverage and breakpoint frequencies at CTCF clusters.
Points represent averages over one of 100 quantiles, and are shaded by strength; black edges indicate quantiles with no observed deletion coverage or breakpoints, plotted at the minimal y-value, for display.

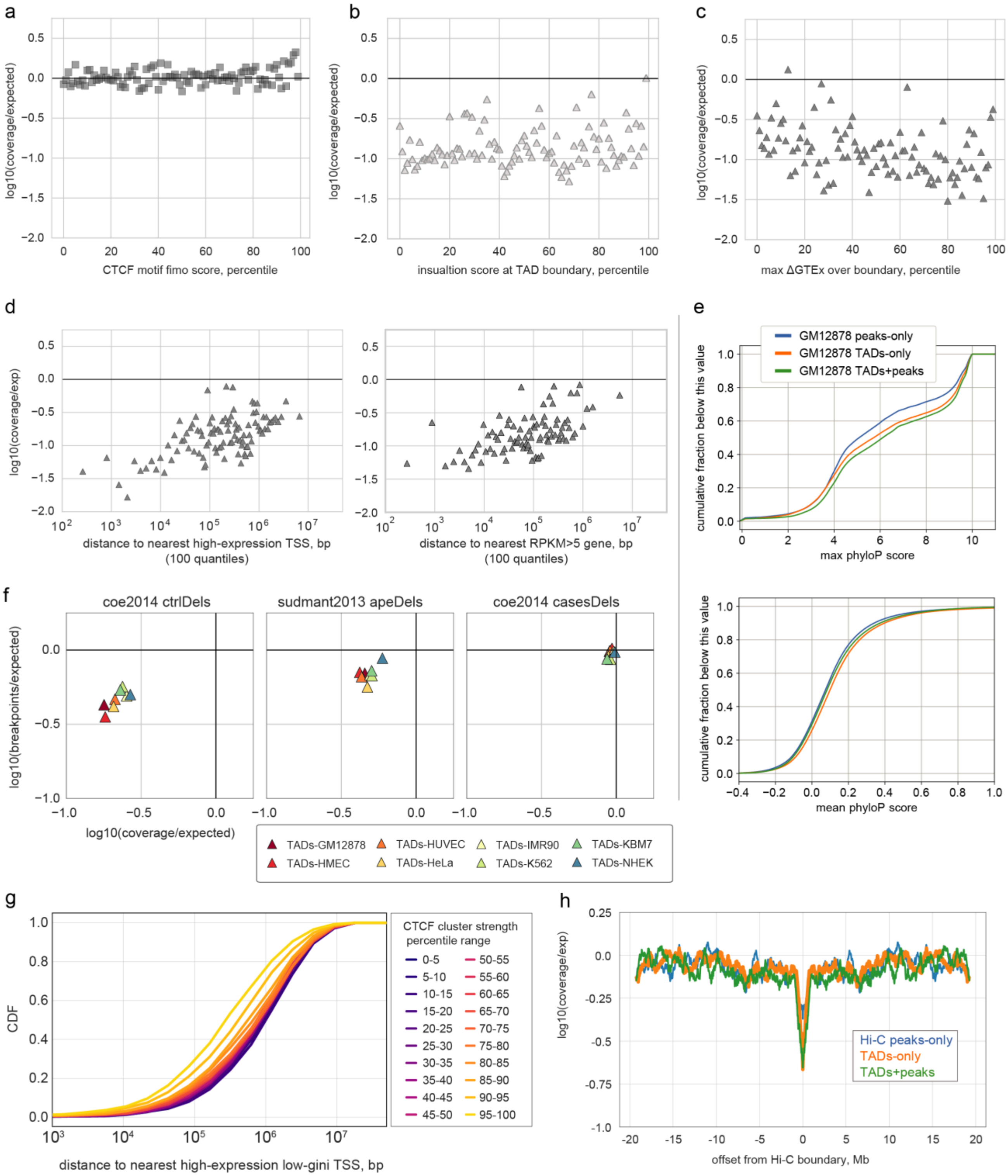

Fig. S3. (caption on next page)

Fig. S3.
a. Deletion coverage at CTCF motifs, stratified by CTCF fimo motif quality (9) for 100 quantiles.
b. Deletion coverage at TAD boundaries versus their insulation score for 100 quantiles of insulation score, both calculated from GM12878 Hi-C data. Insulation score is quantified by the contact frequency in a 250kb sliding window along the genome, as previously (10); note this within-cell type score differs greatly from the cross-cell-type measures of CTCF cluster strength and TSS strength used elsewhere.
c. Deletion coverage at TAD boundaries versus the change in GTEx expression over these boundaries for 100 quantiles. The change in GTEx expression was calculated by: summing together expression for TSSs in the 100kb upstream or downstream of each TAD boundary for each tissue type, taking the absolute value of difference between the upstream and downstream values, converting this to ranks on a per-tissue-type basis, and then, as a deletion in the germline could affect expression in any tissue, taking the maximum across tissues of this change in expression.
d. *Left*: Deletion coverage at TAD boundaries versus distance to the nearest highly-expressed TSS (top 10th percentile in GTEx, corresponding to an average >9 RPKM across tissues). This shows an additional depletion at short distances, which then levels out to the genome-wide average depletion at TAD boundaries (~ -0.7 for distances >200kb). ***Right***: similarly, for RPKM >5 and distance to nearest gene start or end (average ~0.6 for distances > 200kb).
e. *top:* Cumulative distribution of maximum phyloP score in the 10kb window defined by the indicated feature; these three categories were obtained by intersecting the set of TAD domain boundaries and Hi-C peak bases from GM12878, and classifying a region as TAD-only, peak-only, or shared. ***bottom:*** Cumulative distributions of mean phyloP score in the same regions. These plots demonstrate that basewise conservation is congruent with deletion frequency in healthy humans for these three categories of chromatin feature.
f. Deletion coverage versus breakpoint frequency for TAD boundaries across cell types for the indicated datasets. Healthy human and ape deletions are consistently depleted across cell types at TAD boundaries, patients with developmental delay and autism consistently show no depletion for either breakpoint frequency or coverage.
g. Cumulative distributions of the distance between a CTCF cluster and the nearest broadly and highly-expressed TSSs (below 10th percentile Gini index, and above 90th percentile aggregate expression), stratified by CTCF cluster strength (20 quantiles). Note that strongly bound CTCF sites across ENCODE cell types are closer to broadly- and highly-expressed TSSs characterized by GTEx, as indicated by the leftward shift of the CDF.
h. A zoomed-out view of the same data as in **Fig. 3F**, showing that all curves approach zero at ~5-10Mb.

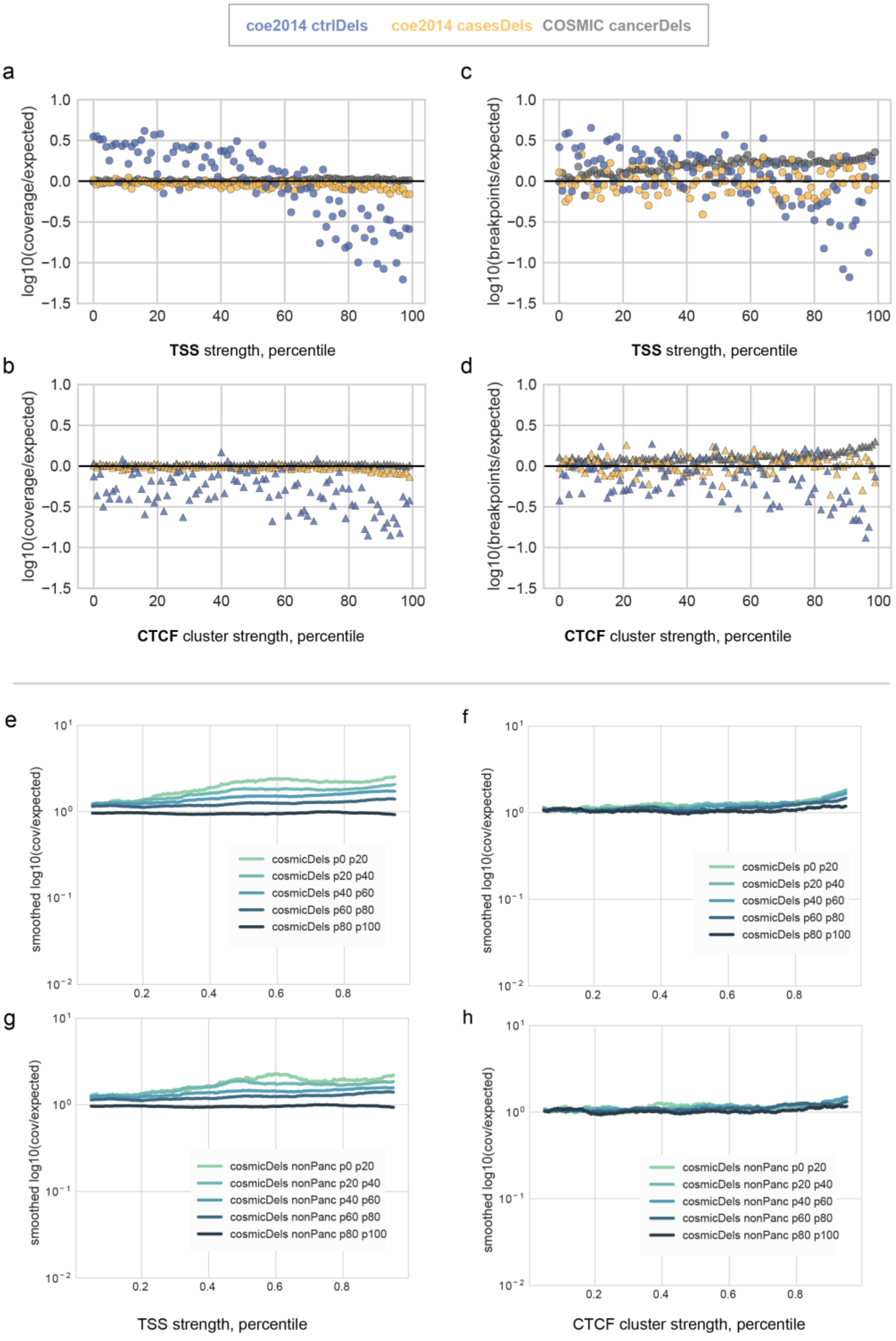

Fig. S4. (caption on next page)

Fig. S4.
a. Deletion coverage is relatively flat as a function of TSSs strength when considering all deletions across all cancer types in COSMIC. Points represent averages for 100 quantiles.
b. Deletion coverage is also relatively flat as a function of CTCF cluster strength
c. Deletion breakpoint frequency, however, is highest for the most active TSSs for COSMIC deletions (blue), opposite the trend in healthy humans (green), and distinct from the apparent lack of a trend for deletions in patients with developmental delay and autism (yellow).
d. Deletion breakpoint frequency shows a similar trend for CTCF cluster strength.
e. Deletion coverage, stratified by deletion length shows short deletions have a greater dependence on TSSs strength, as expected, and explaining the difference between coverage and breakpoint frequency in **a-d**. Averages plotted with a sliding window (+/-5 percentiles).
f. A similar pattern is seen as a function of CTCF cluster strength.
g,h. As pancreatic cancers contributed roughly half of the deletions in COSMIC, we re-examined the above two trends excluding pancreatic deletions, and saw similar patterns for TSSs and CTCF clusters (as in **e,f**).

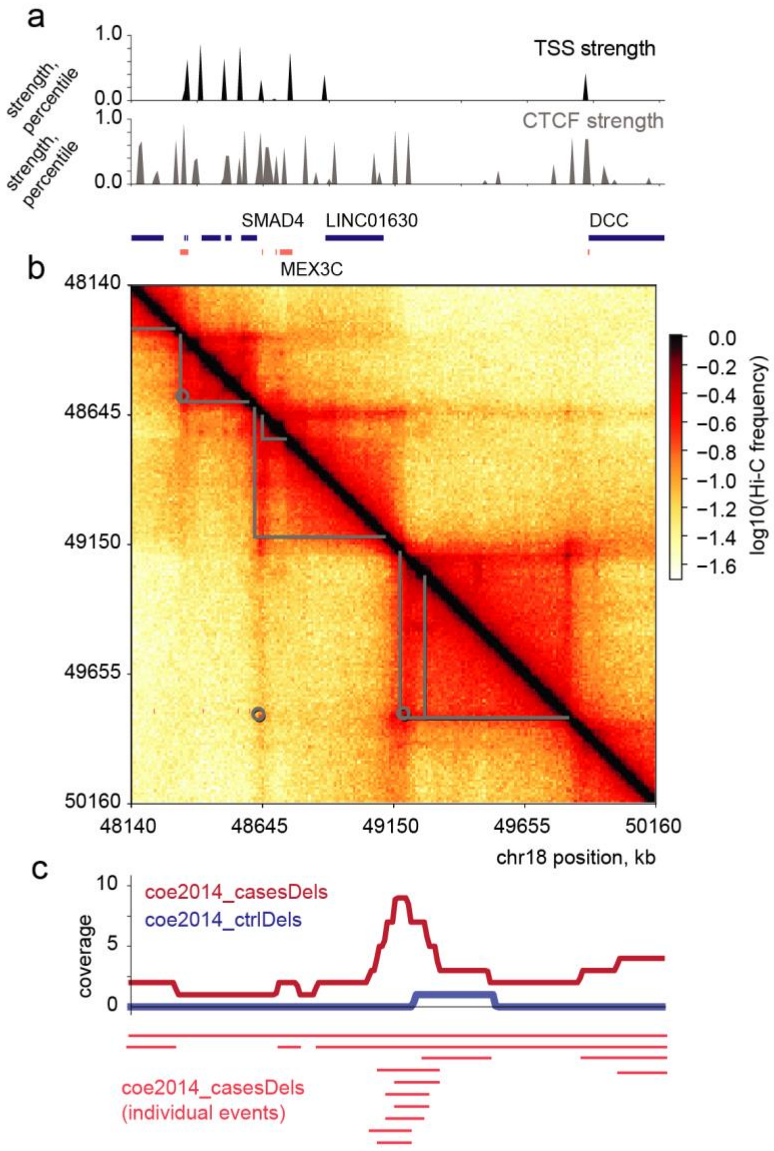

Fig. S5:
Focal enrichment of deletions in cases at a TAD boundary on chr18.
A: (*top*) 10kb binned profiles of TSS and CTCF cluster strength in this region, (*bottom*) positions of genes colored by orientation (blue, forward; red, reverse).
B: Hi-C map for this region from GM12878 cells at 10kb resolution (Rao et al., 2014), with associated TAD and Hi-C peak calls overlaid as grey lines and circles.
C: Coverage of deletions in patients (cases, red) and controls (blue) over this region; red bars below show individual events in patients that build up this coverage profile.

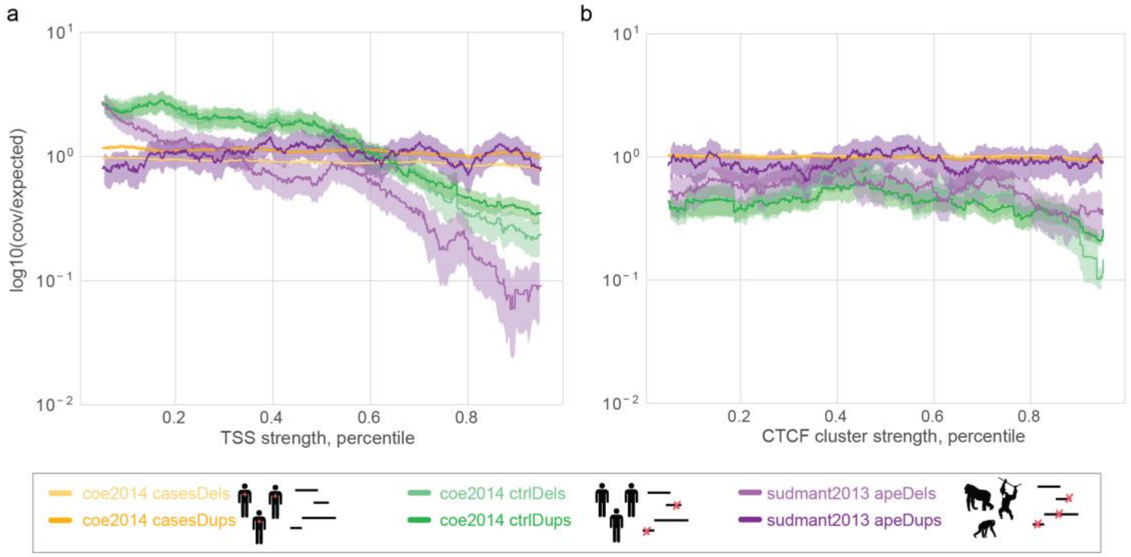

Fig. S6.
a. For deletions, strong TSSs are most avoided for apes, then for healthy humans, and are not avoided in developmental delay patients, suggesting that these disease-associated deletions may be deleterious. Duplications show less avoidance of TSSs than deletions in healthy humans, and no avoidance for ape duplications. Curves show average expected coverage as a function of TSS strength in a sliding window (+/-5 percentiles). Areas represent 5th & 95th percentiles of sliding mean calculated over 1000 bootstrap samples.
b. CTCF clusters are depleted for deletions across CTCF cluster strengths in both apes and healthy humans, consistent with their disruption being generally deleterious and under purifying selection. As at TSSs, ape duplications display no preferential avoidance of CTCF clusters.

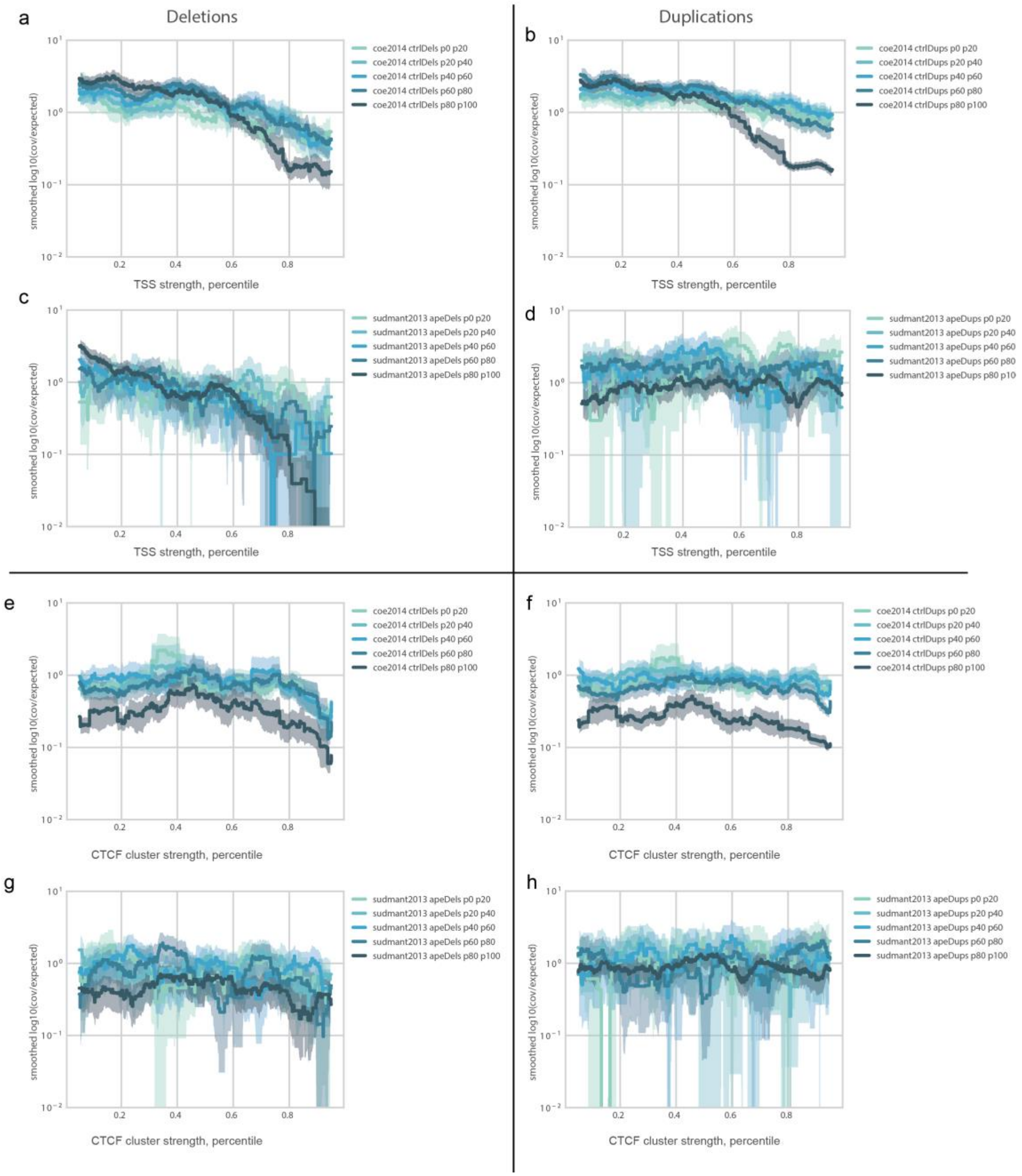

Fig. S7.
Coverage for deletions (**a,c,e,g**) and duplications (**b, d, f, h**), versus total expression (**a-d**) or CTCF strength (**e-h**), additionally stratified by variant length (five quantiles), for the indicated datasets. Sliding window and bootstraps as in **Fig. S6**. Note that the longest duplications are the main contributor to the avoidance of active TSSs and strongly bound CTCF sites observed for healthy human variants.

Dataset S1. GO-rilla enrichments for TSSs around TAD boundaries that were significantly deleted in the ape, healthy human, and patients with developmental disease or autism datasets. All three sets of terms were calculated relative to the set of all TSSs +/-500kb around TAD boundaries using the 'two unranked lists of genes' running mode (http://cbl-gorilla.cs.technion.ac.il/).

Dataset S2. Input lists for GO-rilla enrichment calculations (three target sets and the background set).

Dataset S3. Sheet 1: Statistics of variants for indicated datasets. The unique_variants column for (Sudmant et al., 2013) ape variants indicates the number of parsimonious variants relative to the human genome (i.e. those not better explained by an alteration in the human lineage). For other datasets, this column indicates the number of variants with unique start and endpoints. For all datasets, unique_variants includes only autosomal variants. **Sheet 2:** sources for curated chromatin features.

	Deleted regions		Coverage peaks	
	Overlap	NonOverlap	Overlap	NonOverlap
Cases	12517	74616	25	151
Controls	5905	51810	25	356

Table S1. Left: Table for significantly deleted regions in cases and controls, and whether they overlap TAD boundaries. **Right.** Table for peaks in 10kb binned coverage profiles in cases and controls, and whether they overlap TAD boundaries.

References

1. Weischenfeldt J, et al. (2017) Pan-cancer analysis of somatic copy-number alterations implicates IRS4 and IGF2 in enhancer hijacking. *Nat Genet* 49(1):65–74.
2. Coe BP, et al. (2014) Refining analyses of copy number variation identifies specific genes associated with developmental delay. *Nat Genet* 46(10):1063–1071.
3. Eden E, Navon R, Steinfeld I, Lipson D, Yakhini Z (2009) GOrilla: a tool for discovery and visualization of enriched GO terms in ranked gene lists. *BMC Bioinformatics* 10:48.
4. Dong D, He G, Zhang S, Zhang Z (2009) Evolution of Olfactory Receptor Genes in Primates Dominated by Birth-and-Death Process. *Genome Biol Evol* 1:258–264.
5. Beroukhim R, et al. (2010) The landscape of somatic copy-number alteration across human cancers. *Nature* 463(7283):899–905.
6. Eden E, Lipson D, Yogev S, Yakhini Z (2007) Discovering Motifs in Ranked Lists of DNA Sequences. *PLOS Comput Biol* 3(3):e39.
7. Rao SSP, et al. (2014) A 3D map of the human genome at kilobase resolution reveals principles of chromatin looping. *Cell* 159(7):1665–1680.
8. Gordon D, et al. (2016) Long-read sequence assembly of the gorilla genome. *Science* 352(6281):aae0344.
9. Grant CE, Bailey TL, Noble WS (2011) FIMO: scanning for occurrences of a given motif. *Bioinformatics* 27(7):1017–1018.
10. Nora EP, et al. (2017) Targeted Degradation of CTCF Decouples Local Insulation of Chromosome Domains from Genomic Compartmentalization. *Cell* 169(5):930-944.e22.
